# Assessment of a multiple biomarker panel for diagnosis of amyotrophic lateral sclerosis

**DOI:** 10.1186/s12883-016-0689-x

**Published:** 2016-09-15

**Authors:** Xueping Chen, Yongping Chen, Qianqian Wei, Ruwei Ou, Bei Cao, Bi Zhao, Hui-Fang Shang

**Affiliations:** Department of Neurology, West China Hospital, Sichuan University, Chengdu, Sichuan China

**Keywords:** Amyotrophic lateral sclerosis, pNfH, S100-β, cystatin C, CHIT, Biomarker

## Abstract

**Background:**

The aim of the study was to assess a panel of promising biomarkers for their ability to improve diagnosis of sporadic amyotrophic lateral sclerosis (ALS).

**Methods:**

Forty patients with sporadic ALS and 40 controls with other neurological diseases were evaluated. Levels of phosphorylated neurofilament heavy chain (pNfH), S100-β, cystatin C, and chitotriosidase (CHIT) in cerebrospinal fluid were assayed using two-site solid-phase sandwich ELISA.

**Results:**

Patients with sporadic ALS showed higher levels of pNfH and CHIT than controls, but lower levels of cystatin C. Multivariate logistic regression that adjusted for patient age and sex identified significant associations between sporadic ALS and levels of pNfH, CHIT and cystatin C. Levels of pNfH correlated positively with rate of progression and decline based on the Amyotrophic Lateral Sclerosis Functional Rating Scale - Revised. Based on receiver operating curve analysis, a pNfH cut-off of 437 ng/L discriminated patients from controls with a sensitivity of 97.3 % and specificity of 83.8 %. A CHIT cut-off of 1593.779 ng/L discriminated patients from controls with a sensitivity of 83.8 % and specificity of 81.1 %. Combining the two biomarkers gave a sensitivity of 83.8 % and specificity of 91.9 %.

**Conclusions:**

Levels of pNfH in cerebrospinal fluid may be a reliable biomarker for diagnosing ALS, and combining this biomarker with levels of CHIT may improve diagnostic accuracy.

## Background

Amyotrophic lateral sclerosis is a fatal neurodegenerative disorder. It is diagnosed based on purely on clinical evaluation and differential diagnosis to exclude other possible conditions, highlighting the need to identify objective biomarkers that may aid diagnosis and perhaps help predict progression and prognosis.

Several candidate ALS biomarkers have been identified in cerebrospinal fluid (CSF), including neurofilament proteins, S100-β, cystatin C, and chitotriosidase (CHIT) [1, 2]. The validity of these biomarkers remains controversial, since they were identified using targeted approaches rather than unbiased screens and they have been assessed individually in separate studies, rather than in parallel. In addition, studies have not assessed the diagnostic potential of combinations of these candidate biomarkers.

Here we examined several candidate ALS biomarkers: phosphorylated neurofilament heavy chain (pNfH), S100-β, cystatin C, and CHIT. Our objective was to identify, in parallel comparisons, which biomarker(s) may be the most effective, as well as assess the diagnostic potential of biomarker combinations.

## Methods

### Patients and samples

This study involved 40 patients clinically diagnosed with definite or probable sporadic ALS between May 2006 and November 2013 in the Department of Neurology at West China Hospital, Sichuan University (Chengdu, China). We also recruited 40 age- and sex- matched control patients diagnosed with non-ALS neurological diseases. These controls had lower motor neuron disease (*n* = 21), including spinal muscular atrophy (*n* = 8), multifocal motor neuropathy with conduction block (*n* = 6), spinal and bulbar muscular atrophy (*n* = 4), and chronic inflammatory demyelinating neuropathy (*n* = 3); or upper motor neuron disease (*n* = 19), including cervical myelopathy (*n* = 9), multiple sclerosis (*n* = 7), and hereditary spastic paraparesis (*n* = 3).

Motor and functional status of patients with sporadic ALS was quantified by neurologists using the Amyotrophic Lateral Sclerosis Functional Rating Scale - Revised (ALSFRS-R). ‘Progression rate’ was calculated from the equation: [(48 − ALSFRS-R score at baseline evaluation)/disease duration in months from symptom onset to evaluation]. Annual decline in ALSFRS-R was calculated from the equation: [(value at baseline examination − value at last examination)/years between the two examinations]. Monthly decline in ALSFRS-R was calculated by dividing the annual decline in ALSFRS-R by 12.

CSF samples were obtained from all study participants by lumbar puncture. Samples were centrifuged at 3000 rpm for 10 min at 4 °C to remove particulate matter. Protease inhibitor cocktail was added, and samples were aliquoted and stored at −80 °C until analysis.

### ELISA

Commercial two-site solid-phase sandwich ELISAs were used to assay levels of pNfH (RD191138300R, Biovendor), S100-β (RD192090100R, Biovendor), cystatin C (RD191009100, Biovendor) and CHIT (cy-8074, MBL) in CSF samples. Kits were used according to the manufacturers’ instructions. Before definitive measurements were taken, we assessed the stability of all four biomarkers in CSF by assaying their levels in samples that had been collected less than 30 min previously or more than 24 h previously and then stored at different temperatures. Biomarker levels remained stable after storage at −80 °C. Biomarkers did not appreciably aggregate under sample storage and thawing conditions, based on recovery experiments in which known amounts of biomarkers were added to the sample.

### Statistical analysis

Data were reported as mean ± standard deviation or median (range) and analyzed using SPSS 17.0 (IBM, Chicago, IL, USA). Since most data showed a skewed distribution, intergroup differences were assessed for significance using non-parametric statistical tests, i.e., the Mann–Whitney U and Kruskal–Wallis tests, followed by pair-wise post hoc tests corrected for multiple comparisons. The threshold of significance was defined as *p* < 0.05.

Possible associations of sporadic ALS with levels of pNfH, S100-β, cystatin C, and CHIT in CSF were explored using multivariate logistic regression, with adjustment for age and sex as potential confounders. In this regression, biomarker levels were treated as categorical variables by stratification into quartiles based on the distributions observed in controls. The quartile with the lowest biomarker levels served as the reference group. Receiver operating curve (ROC) analysis was used to calculate an optimal cut-off level to discriminate patients with sporadic ALS from controls. Spearman rank correlation was used to explore possible correlations between demographic variables, disease duration, ALSFRS-R and progression rate on one hand and biomarker levels on the other. Patients were divided into various subgroups, and biomarker levels were compared between them using the Van Elteren test.

## Results

Demographic and clinical characteristics of patients and controls are listed in Table 1. Most patients (33) had the spinal-onset form of the disease. Among all patients, mean rate of disease progression, in terms of ALSFRS-R score, was 0.91/month. Twelve patients were defined as showing rapid progression because their monthly rate of progression was >0.91. Among all patients, mean rate of decline, again in terms of ALSFRS-R score, was 11.01/year, corresponding to a mean rate of decline of 0.92/month. Ten patients were defined as showing rapid worsening because their monthly rate of decline was >0.92. Eleven patients died during follow-up, and three were lost to follow-up.

**Table 1:** Demographic and clinical characteristics of patients with sporadic amyotrophic lateral sclerosis and controls with non-ALS neurological disorders

Characteristic	ALS (*n* = 40)	Controls (*n* = 40)
Male, n (%)	26 (55)	24 (60)
Age, yr	52.08 ± 11.45	53.68 ± 11.93
Age at onset, yr	57.96 ± 10.96	
Disease duration, mo	17.61 (2.77–50.43)	
Site of onset (spinal/bulbar), n	33/7	
ALSFRS-R score	38.32 (21–47)	
Progression rate	0.91 (0.08–5.06)	
Rapidly progressive (progression rate > 0.91/month)/Slowly progressive (progression rate ≤ 0.91/month)	12/28	
Annual decline of ALSFRS	11.01 (3.10–33.31)	
Monthly decline of ALSFRS	0.92 (0.26–2.78)	
Rapidly worsening 0.92/month)/	10/16	
Slowly worsening (monthly decline of ALSFRS-R ≤0.92/month)	26/11/3	
Survival/death/ lost to follow-up	26/11/3	

Values shown are n, n (%), mean ± SD, or median (range)

ALSFRS-R, Amyotrophic Lateral Sclerosis Functional Scale - Revised

### Levels of pNfH, CHIT, cystatin C, and S100-β in CSF

**Fig. 1:**
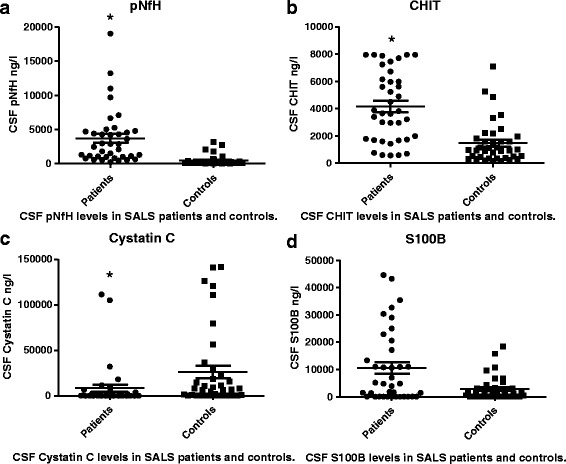
Scatter plot of levels of pNfH, S100-β, cystatin C, and CHIT in CSF in patients with sporadic ALS and control patients with non-ALS neurological disorders. *the difference is significant

Patients with sporadic ALS showed significantly higher levels of pNfH and CHIT in CSF than controls did (*P* < 0.0001; Fig. 1a-b). Conversely, patients with ALS showed a significantly lower level of cystatin C (*P* = 0.0009; Fig. 1c). Patients and controls did not differ significantly in levels of S100-β (Fig. 1d).

### Associations of sporadic ALS with levels of pNfH, CHIT, cystatin C, or S100-β

Multivariate logistic regression was used to explore possible associations of ALS status with levels of pNfH, S100-β, cystatin C, or CHIT in CSF. The regression model adjusted for age and sex as possible confounders. Sporadic ALS was significantly associated with higher levels of pNfH, higher levels of CHIT, and lower levels of cystatin C. No significant correlation was identified between disease status and levels of S100-β.

**Fig. 2:**
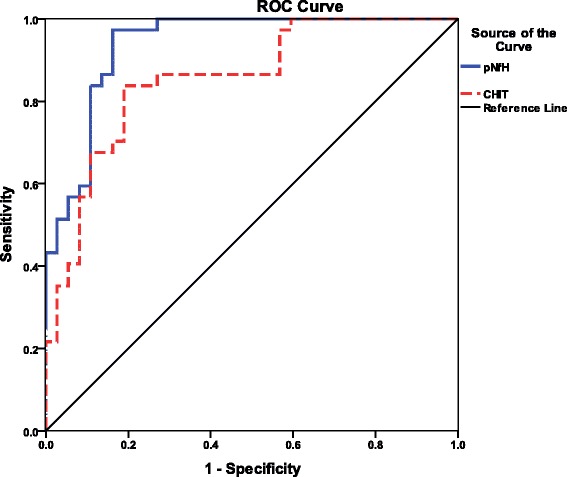
Receiver operating curve (ROC) analysis

Using ROC analysis, we calculated an optimal pNfH cut-off of 437 ng/L to discriminate patients with sporadic ALS from controls. This cut-off showed a sensitivity of 97.3 % and specificity of 83.8 %; the area under the ROC curve (AUC) was 0.938 (95 % CI 0.884–0.991). Using a CHIT cut-off of 1593.779 ng/L gave a sensitivity of 83.8 %, specificity of 81.1 % and AUC of 0.854 (95 % CI 0.768–0.940; Fig. 2). Simultaneously applying both cut-offs gave a sensitivity of 83.8 %, specificity of 91.9 % and AUC of 0.952 (95 % CI 0.909–0.994.

### Associations of clinico-demographic characteristics with levels of pNfH, CHIT, and cystatin C

Subgroups of patients with bulbar or spinal onset of sporadic ALS were similar in terms of pNfH, CHIT, and cystatin C levels. Levels of pNfH were significantly higher in patients with rapidly progressing disease than in those with slowly progressing disease (*P* = 0.001), and they were higher in patients with rapidly worsening disease than in those with slowly worsening disease (*P* = 0.0143). Levels of CHIT and cystatin C did not differ significantly between subgroups stratified by monthly rate of progression or decline. No significant associations were observed between ALSFRS-R score or disease duration on one hand and levels of the three biomarkers on the other (Table 2).

**Table 2:** Comparison of levels of pNfH, CHIT, and cystatin C in cerebrospinal fluid between subgroups of patients with sporadic ALS

Subgroup	pNfH (ng/L)	*P* value	CHIT (ng/L)	*P* value	cystatin C (ng/L)	*P* value
Site of onset						
Spinal	4676 (238–19002)	0.43	4457 (572–7945)	0.08	10330 (62–111466)	0.35
Bulbar	3022 (552–5265)		2886 (91–7945)		3065 (119–1113)	
Progression rate						
>0.91/month	5969 (996–19002)	0.001	3927 (572–7945)	0.85	6566 (62–105031)	0.80
≤0.91/month	1969 (238–5030)		3791 (91–7945)		12900 (62–111466)	
Worsening rate						
>0.92/month	6171 (1309–19002)	0.0143	4668 (598–7945)	0.67	5188 (78–32186)	0.83
≤0.92/month	2670 (238–13223)		4243 (572–7945)		15610 (62–111466)	

*ALS* amyotrophic lateral sclerosis, *CHIT* chitotriosidase, *pNfH* phosphorylated neurofilament heavy chain

## Discussion

In this study, we found that levels of pNfH in CSF were significantly higher in patients with sporadic ALS than in controls with non-ALS neurological disorders. Similar results have been reported in other populations [3, 4]. In fact, Ganesalingam et al. reported 10-fold higher mean levels of pNfH in CSF in patients with ALS [5]. A large study of 455 patients reported significantly higher levels of pNfH in patients than in controls [6]. Multivariate regression of our data showed that sporadic ALS was associated with high levels of pNfH in CSF, even after adjusting for potential confounding variables. This suggests a possible role of pNfH in the pathogenesis of ALS, and it is consistent with studies showing that phosphorylation of NfH slows its axonal transport and interaction with other cytoskeletal proteins, affecting the course of ALS [7]. Higher levels of pNfH in the CSF of patients with ALS may reflect higher content of axonal proteins in motor neurons and greater extent of axonal injury, leading to the release of large amounts of pNfH into the extracellular space and ultimately into the CSF [8]. A recent study investigated CSF of 455 patients for pNfH, and it also reported that pNfH levels were significant higher in ALS patients compared to the control groups [6].

Building on this observed association between sporadic ALS and levels of pNfH, we examined whether this biomarker shows diagnostic potential. Using a cut-off of 437 ng/L to discriminate between patients and controls gave sensitivity of 97.3 % and specificity of 83.8 %. These results are consistent with other studies reporting relatively high sensitivity and specificity, although the optimal cut-off values vary substantially. Using a cut-off of 950 ng/L, Brettschneider et al. reported sensitivity of 71 % and specificity of 88 % [3]. Using a cut-off of 502 ng/L, Reijn et al. obtained sensitivity of 72 % and specificity of 80 % [4]. Steinacker et al. reported 83 % sensitivity and 77 % specificity with a cut-off of 560 ng/L [6]. Ganesalingam et al. reported sensitivity of 87.7 % and specificity of 93.7 % with a cut-off of 635 ng/L [9], while the same laboratory obtained respective values of 90 % and 87 % using a cut-off of 1200 ng/L in a larger patient sample [5]. This large variation in cut-off value may reflect the relatively small size of study populations, ethnic differences among the populations, and the intrinsic heterogeneity of sporadic ALS. The large variation may also reflect differences among the ELISA kits used. Given the strong evidence of diagnostic potential for pNfH levels in CSF, future work is urgently needed to develop and validate standard clinical biochemistry procedures for assaying this biomarker [10].

While pNfH levels are associated with presence of sporadic ALS, they do not appear to predict site of disease onset. In our study, levels were similar between subgroups of patients with bulbar or spinal onset. This is consistent with previous studies [3, 11].

We also investigated whether pNfH levels in CSF may show prognostic potential. We found that these levels did not correlate with clinical disability (ALSFRS-R score) at the time of diagnosis, consistent with a previous study [3]. However, these levels were significantly higher in our patients who suffered more rapid decline in ALSFRS-R score. In addition, the levels were significantly higher in patients with more rapidly progressive disease based on the progression rate [12, 13]. This echoes previous work associating elevated levels of pNfH with faster progression of ALS [3]. This suggests that pNfH levels may predict functional worsening in ALS. It may be that more aggressive disease is associated with increased, sustained cytoskeletal breakdown within motor neurons, which would lead to greater leakage of pNfH into CSF.

Our data also showed CHIT, an enzyme synthesized by microglia or infiltrating macrophages, to be present at significantly higher levels in CSF in patients with sporadic ALS than in controls. This is consistent with previous studies [1, 2]. Higher CHIT levels may reflect a neuroinflammatory response triggered by microglia, since CHIT is considered a marker of microglial activation in stroke [14] and, like pro-inflammatory cytokines, an index of inflammation severity [15]. We further found that CHIT levels can distinguish patients from controls with sensitivity of 83.8 % and specificity of 81.1 %, giving an acceptable AUC of 0.854. To our knowledge, this is the first evidence that CHIT has diagnostic potential in sporadic ALS. At the same time, we found similar CHIT levels in subgroups of patients with rapidly or slowly progressing disease, in contrast to a previous study [2]. This may reflect clinical heterogeneity among patients with sporadic ALS.

We examined whether the combination of pNfH and CHIT levels may provide greater diagnostic accuracy than either biomarker alone. Using both gave a much higher specificity than pNfH alone (91.9 % vs. 83.8 %), but a lower sensitivity (83.8 % vs. 97.3 %). Our results suggest that combining pNfH and CHIT improves diagnostic specificity without sacrificing too much sensitivity.

We found that levels of cystatin C in CSF were significantly lower in patients with sporadic ALS than in controls. While several studies have reported a similar decrease in cystatin C levels in patients [16–19], analysis of samples from six centers in Europe failed to detect differences between patients and controls [20]. This discrepancy may reflect well-known preanalytic artifacts in assaying cystatin C [21]. It may also reflect complex roles of cystatin C: this cysteine proteinase inhibitor has been implicated in both neuronal degeneration and neuronal repair. For example, Wilson et al. reported that cystatin C levels increase when ALS remains stable or progresses slowly, whereas levels decrease when disease progresses rapidly [22]. However, our data failed to indicate an association of cystatin C levels with disease progression, duration, or severity. Moreover, cystatin C levels could not distinguish patients from controls. Future studies should examine in greater detail the potentially complex role(s) of cystatin C in ALS.

We found no association between sporadic ALS and levels of S100-β in CSF. This contrasts with several previous studies suggesting that this protein, which modulates the activity of effector proteins or cells, is present at higher levels in the CSF of patients with ALS [23–25]. Regardless of whether ALS involves changes in S100-β, assaying this protein is unlikely to be helpful for differential diagnosis of the disease, since levels of the protein have also been shown to change in other neurodegenerative disorders.

The results from our study should be interpreted with caution in light of at least three substantial limitations. One is our small sample, and another is the cross-sectional design. Future work should monitor biomarker levels and disease onset and progression longitudinally. Third, our assays were not independently verified by an external laboratory.

Despite these limitations, the present study strengthens the evidence base that pNfH and CHIT are neuropathological hallmarks of ALS. Levels of pNfH and CHIT in CSF were significantly higher in patients than in controls, while levels of cystatin C were significantly lower in patients. In addition, pNfH levels in CSF correlated with disease severity and progression, and they discriminated patients from controls with high sensitivity and specificity. These findings point to pNfH as perhaps the single most promising candidate biomarker for ALS. At the same time, combining pNfH with CHIT may improve diagnostic accuracy. Given the heterogeneity of ALS, our results highlight the need for large longitudinal studies of patients with several disease phenotypes in order to verify and compare the ability of these biomarkers, alone and in combination, to differentiate ALS from related disorders.

## Conclusions

Despite these limitations, the present study strengthens the evidence base that pNfH and CHIT are neuropathological hallmarks of ALS. Levels of pNfH and CHIT in CSF were significantly higher in patients than in controls, while levels of cystatin C were significantly lower in patients. In addition, pNfH levels in CSF correlated with disease severity and progression, and they discriminated patients from controls with high sensitivity and specificity. These findings point to pNfH as perhaps the single most promising candidate biomarker for ALS. At the same time, combining pNfH with CHIT may improve diagnostic accuracy. Given the heterogeneity of ALS, our results highlight the need for large longitudinal studies of patients with several disease phenotypes in order to verify and compare the ability of these biomarkers, alone and in combination, to differentiate ALS from related disorders.

## Abbreviations

ALS: Amyotrophic lateral sclerosis
ALSFRS-R: Amyotrophic Lateral Sclerosis Functional Rating Scale - Revised
AUC: Area under the ROC
CHIT: Chitotriosidase
CSF: Cerebrospinal fluid
pNfH: Phosphorylated neurofilament heavy chain
ROC: Receiver operating curve
